# Plasma Biomarkers for Detecting Hodgkin's Lymphoma in HIV Patients

**DOI:** 10.1371/journal.pone.0029263

**Published:** 2011-12-15

**Authors:** Susan M. Varnum, Bobbie-Jo M. Webb-Robertson, Nancy A. Hessol, Richard D. Smith, Richard C. Zangar

**Affiliations:** 1 Department of Cell Biology and Biochemistry, Pacific Northwest National Laboratory, Richland, Washington, United States of America; 2 Department of Computational Biology and Bioinformatics, Pacific Northwest National Laboratory, Richland, Washington, United States of America; 3 Department of Biological Sciences Division, Pacific Northwest National Laboratory, Richland, Washington, United States of America; 4 Department of Clinical Pharmacy, University of California at San Francisco, San Francisco, California, United States of America; Food and Drug Administration, United States of America

## Abstract

The lifespan of people with human immunodeficiency virus (HIV) infection has increased as a result of effective antiretroviral therapy, and the incidences of the AIDS-defining cancers, non-Hodgkin's lymphoma and Kaposi sarcoma, have declined. Even so, HIV-infected individuals are now at greater risk of other cancers, including Hodgkin's lymphoma (HL). To identify candidate biomarkers for the early detection of HL, we undertook an accurate mass and elution time tag proteomics analysis of individual plasma samples from either HIV-infected patients without HL (controls; n = 14) and from HIV-infected patient samples with HL (n = 22). This analysis identified 60 proteins that were statistically (p<0.05) altered and at least 1.5-fold different between the two groups. At least three of these proteins have previously been reported to be altered in the blood of HL patients that were not known to be HIV positive, suggesting that these markers may be broadly useful for detecting HL. Ingenuity Pathway Analysis software identified “inflammatory response” and “cancer” as the top two biological functions associated with these proteins. Overall, this study validated three plasma proteins as candidate biomarkers for detecting HL, and identified 57 novel candidate biomarkers that remain to be validated. The relationship of these novel candidate biomarkers with cancer and inflammation suggests that they are truly associated with HL and therefore may be useful for the early detection of this cancer in susceptible populations.

## Introduction

In the presence of human immunodeficiency virus (HIV) infection, non-Hodgkin's lymphoma (NHL) and Kaposi sarcoma (KS) were the first malignancies used to define acquired immune deficiency syndrome (AIDS). People with HIV infection are also at increased risk of a number of other cancers [1]. As people with HIV infection live longer due to highly active antiretroviral therapy (HAART), the incidence of these non-AIDS-defining cancers has increased among HIV-infected individuals. One of the most common of these malignancies is Hodgkin's lymphoma (HL), and it has been estimated that people with HIV/AIDS have a 5.6- to 14.7-fold increased risk of developing HL compared to the general population [1]–[3].

HL is a solid tumor that is comprised of no more than 2% of the cancerous B lymphocytes. Instead, these lymphocytes secrete a wide range of cytokines that attract numerous normal leukocytes that then comprise the large majority of the tumor [4]. Thus, HL is largely seen as an uncontrolled inflammatory disease [4]. In people with immunosuppression, HL is believed to result from the Epstein-Barr virus (EBV). EBV is present in nearly all adults, but typically only causes HL when the immune system is suppressed, such as with HIV infection [4].

The increase in non-AIDS-defining cancers has created a greater need for the early detection of these malignancies in this susceptible population. It seems likely that HIV-infected individuals would benefit from a routine, non-invasive screen for HL, but no such screen exists. Rather, HL patients are identified after they become symptomatic [5]. Chemotherapy and radiation therapy have been shown to be very effective at causing HL remission, but morbidity and mortality associated with these treatments is substantial [6]. Early-stage HL is generally treated less intensively, suggesting that early detection of HL would result in less treatment-related toxicity. Cancer treatment strategies for HIV-infected individuals with HL are the same as for non-AIDS subjects [6], but HIV-infected patients require additional vigorous supportive care (HAART, antifungals, neutrophil-simulating growth factors).

To identify candidate biomarkers for HL detection, we analyzed plasma samples from HIV-infected patients, with or without HL, using accurate mass tag and time (AMT) tag proteomics, and thereby identified a set of 60 proteins. As a group, these proteins are associated with both cancer and inflammation and therefore are promising candidate biomarkers for early detection of HL.

## Materials and Methods

### Ethics Statement

The study protocol was approved by the George Washington University Medical Center Institutional Review Board. Written informed consent was obtained from all study participants. Additional approval from the PNNL Institutional Review Board, which included a review of the George Washington University Medical Center IRB approval, was obtained before samples were transferred to PNNL.

### Human Subjects and Sample Processing

Frozen, human plasma samples were provided by the AIDS and Cancer Specimen Resource (San Francisco, CA). The control subjects (HIV-infected without HL) were chosen to approximately match the cases (HIV-infected with HL) based on gender and age (Table 1). In most cases, plasma samples were collected from HIV-infected subjects with HL that had not received HL chemotherapy within at least 30 days (n = 12). Information on chemotherapy was unknown for some cases (n = 9) and one sample was known to have been collected within 30 days of chemotherapy. For all processing and analytical steps, samples were blocked based on HL status and randomized [7]. The 12 most abundant plasma proteins were depleted using the Proteome Purify™ 12 immunodepletion resin (R&D Systems), according to the manufacturer's protocol. The remaining plasma proteins were precipitated using ice-cold trichloroacetic acid at a final concentration of 10%, followed by overnight incubation at 4°C and centrifugation at 14,000 x *g* for 5 minutes. The pellet was washed with cold acetone and dried at room temperature for 5 minutes prior to suspension in 25 µL of 100 mM ammonium bicarbonate, 8 M urea, 2 M thiourea and 5 mM dithiothreitol, and heating to 60°C for 30 min. The samples were then diluted 4-fold with 100 mM ammonium bicarbonate and CaCl_2_ was added to 1 mM. Methylated, sequencing-grade trypsin (Promega, Madison, WI) was added at a substrate-to-enzyme ratio of 50:1 (mass:mass) and incubated at 37°C for 15 h. Peptides were then purified by binding to a 1 mL SPE C_18_ column (Supelco, Bellefonte, PA) and eluting with 1 mL of methanol. After concentration in a SpeedVac, the samples were dissolved in 25 mM ammonium bicarbonate and frozen at −20°C until analysis.

**Table 1:** Subject characteristics.

	HIV+	HIV+/HL+
**Number**	14	22
**Gender (M/F)**	10/4	20/2
**Age (min-max)**	53.9±5.9^a^ (42–63)	49.2±8.8 (34–66)
**CD4 count** b	404±380	203±224
**CD4 count <200** c	4 (29%)	12 (55%)
**HAART** d **(yes, no, unknown)**	12, 1, 1	11, 4, 7
**Lymph node involvement**	NA^e^	6

a Mean ± standard deviation.

b Units are CD4-positive cell counts per µL blood.

c CD4 counts of less than 200/µL has been used to define the presence of AIDS in HIV positive subjects.

d Indicates current usage of HAART antiretroviral therapy at the time of the blood draw. “Unknown” indicates that HAART usage at the time of the blood draw is unknown. Prior usage is unknown for all subjects.

e NA, not applicable.

### Liquid Chromatography-Mass Spectrometry

Peptide samples were analyzed using a custom-built, automated, high-pressure nanocapillary liquid chromatography (HPLC) system [8] coupled on-line to a LTQ-Orbitrap mass spectrometer (Thermo Fisher Scientific). The reversed-phase capillary column was prepared by slurry packing 3-µm Jupiter C18 bonded particles (Phenomenex, Torrence, CA) into a 65-cm-long, 75-µm-inner diameter fused silica capillary (Polymicron Technologies, Phoenix, AZ). The mobile phase consisted of 0.2% acetic acid and 0.05% trifluoroacetate in water (A) and 0.1% trifluoroacetate in 90% acetonitrile, 10% water (B). After loading 5 µL (2.5 µg total) of peptides onto the column, the mobile phase was held at 100% A for 50 min. Exponential gradient elution was performed by increasing the mobile phase composition from 0 to 55% B over 115 min. The HPLC column was coupled to the mass spectrometer by an in-house manufactured electrospray ionization interface [9]. The heated capillary inlet temperature and electrospray voltage were 200°C and 2.2 kV, respectively. Data were acquired for 100 min, beginning 65 min after sample injection (15 min into start of buffer B gradient). Orbitrap spectra (AGC 5×10^5^) were collected from 400–2000 m/z at a resolution of 100,000.

The mass spectra were analyzed using the AMT tag approach [9]. The peak lists for each analysis were matched against a mass tag database containing 30,573 tryptic peptides that were previously identified from LC-MS/MS analyses of human plasma [10]–[12]. An estimate of the false-positive rate for peptide identifications in our analysis, which was ≤6%, was obtained by searching the sequence-reversed database [13].

### Statistics

There were 76 mass spectrometry runs included in the proteomics data, including at least two analyses for each of the 36 samples. A total of 6460 unique peptides were initially identified. The data-analysis process first removed peptides for which there were insufficient identifications to perform a statistical analysis [14], which reduced the peptide number to 3814. Next, the peptide abundance measurements were normalized via regression based on all the peptide signals for that mass spectrometer run and then averaged across replicate runs for each sample. The averaged signal intensities were normalized for each sample based on the median absolute deviation of the signal intensity for all peptides identified in each sample. Finally, the data were subjected to two statistical tests to determine peptides having differential abundance due to either signal intensities or presence-absence. A non-parametric Mann-Whitney test was performed using MatLab 2009 to compare signal intensities. Differences in detection incidence was determined using a modified Chi-square test, called a G-test [14]. Using either test, 647 peptides were identified with a p-value of 0.05 or less.

Normalized peptide abundance values were used for protein inference; the peptide abundance measurements were weighted and combined to calculate the relative protein abundances in the two groups using RRollup within DAnTE (http://ncrr.pnl.gov/software/; reference [15]. Only proteins represented by two or more unique peptides were analyzed. Peptide redundancy was reduced with Protein Prophet [16] and by manually selecting characterized proteins over predicted or hypothetical proteins.

### Ingenuity Pathway Analysis

Differentially regulated proteins were analyzed for common biological functions using Ingenuity Pathway Analysis (IPA; Ingenuity Systems, Mountain View, CA) software. To infer significant biological functions, all proteins that were significantly different between groups and their corresponding ratiometric values (HL+/HL-; minimum of 1.5-fold change) were analyzed by IPA. The significance values for biological function were determined using the right-tailed Fisher's Exact Test.

## Results and Discussion

To identify potential circulating biomarkers for HL in a susceptible population, we used the AMT tag LC-MS approach to compare the plasma proteomes from 36 HIV-infected patients with or without HL. The HIV-infected patients without HL served as the control group to identify protein changes associated with HL. The relatively high-throughput characteristics of this approach enabled duplicate analyses of each of the 36 plasma samples, thereby improving data quality. The subject characteristics are described in Table 1. The proteomics analysis identified 3814 peptides across all of the samples, which corresponded to 629 proteins. Of these 3814 peptides, 647 were significantly different between the two disease states, which corresponded to 152 proteins (Tables S1 and S2). Sixty of these proteins met our criteria of being both significantly different between the two groups and having at least 1.5-fold difference in the normalized signal (Table S3). This protein list included well-known acute-phase inflammatory proteins, including C-reactive protein and serum amyloid proteins. To better identify common biological functions within these 60 proteins, Ingenuity Pathway Analysis (IPA) was utilized. In general, proteins that were associated with immune responses and cancer were highly represented in the list of significantly altered biological functions. Many of the proteins we identified are known to be secreted by white blood cells, such as those that infiltrate HL tumors, or by the liver, an important component of the reticuloendothelial system (Table S3). Similarly, the use of IPA to identify common biological functions across the 60 proteins identified “inflammatory response” and “cancer” (Table 2), suggesting that these proteins have a biological relationship with HL. Since only 1% to 2% of the mass of an HL tumor is composed of the cancerous lymphocytes, it may be that the proteins we identified did not originate from these relatively rare cells. Rather, these proteins may be the product of a systemic inflammatory response associated with HL or due to noncancerous leukocytes that form ∼99% of the tumor mass.

**Table 2:** Top BioFunctions and associated proteins, as defined by Ingenuity Pathway Analysis.

Name	p-value range^a^	Associated proteins^b^
Inflammatory Response	3.0×10^−10^ - 1.1×10^−02^	AHSG, AMBP, APOE, ARHGDIB, B2M, C4A/C4B, C8A, CD14, CFD, CRP, GSN, LUM, MBL2, MCAM, MASP1, MSN, PPBP, PNP, PTGDS, PVR, SAA1, SEMA7A, TIMP1
Cancer	9.2×10^−09^ - 1.1×10^−02^	AHSG, AMPB, APOC1, APOE, ARHGDIB, AZGP1, B2M, C4A/C4B, CD14, CFD, COL6A3, CRP, CST3, EFEMP1, GSN, IGFBP2, KRT10, MCAM, MASP1, PNP, PTGDS, SAA1, SELENBP1, TIMP1, YWHAG

a The p-values reflect the range of all the subcategories that the Ingenuity Pathway Analysis included under the header of Inflammatory Response or Cancer. That is, these two BioFunctions have 39 or 35 subcategories, respectively, and each of these subcategories show significant differences, within the range of p-values shown, between the HIV+ (without HL) and HIV+/HL+ groups.

b Abbreviations are defined in Table S3.

At least three of the proteins we identified have been previously reported to be increased in HL subjects compared to healthy controls, including C-reactive protein [17], beta-2-microglobulin [18], [19] and lactate dehydrogenase [18]. These prior studies examined HL in the general population, and did not focus on HIV-positive subjects. In addition, these studies measured these proteins by immunoassay, providing analytical verification as well. In the current study, the presence of HIV in the study population could be a confounding factor. For example, the HL subjects appeared to have slightly more advanced cases of HIV, on the average, as suggested by a trend (p = 0.07, t-test) towards lower CD4-postive cell counts (Table 1), a result which is consistent with slightly more advanced immunosuppression. Even so, since three of the proteins we identified are increased in HL subjects without HIV, it seems clear that the results for these proteins, at least, are not confounded by differential states of HIV infection. Overall, the present study independently validates three plasma biomarkers for HL and identifies an additional 57 novel plasma proteins that appear to be promising candidate biomarkers based on their relationships with cancer and inflammation. These candidate biomarkers remain to be validated in regards to their association with HL.

## Supporting Information

Table S1 Identified significant peptides
(XLSX)

Table S2 Identified significant proteins
(XLSX)

Table S3 Significantly altered proteins and related biological functions.
(DOCX)
