# Supplementary material for: Plasma Biomarkers for Detecting Hodgkin's Lymphoma in HIV Patients
Source: PLoS One. 2011 Dec 15;6(12):e29263. doi: 10.1371/journal.pone.0029263 (PMC3240653; doi:10.1371/journal.pone.0029263)
Supplement: Table S3 — Significantly altered proteins and related biological functions. (DOCX) [file pone.0029263.s003.docx]

**Table S3. Significantly altered proteins and related biological functions.** Peptides were initially identified by mass spectrometry. Selected biological functions and expression sites associated with the individual proteins were identified by Ingenuity Pathway Analysis (IPA).

| **Protein** | **Gene Symbol** | **#pept/ protein** | **Ratio^a^** | **Biological Function** | **Expressed^b^ in** | |
| --- | --- | --- | --- | --- | --- | --- |
|  |  |  |  |  | **Liver** | **WBC^c^** |
| 14-3-3 protein gamma | YWHAG | 4 | 0.59 | Cancer |  | X |
| Actin, alpha skeletal muscle | ACTA1 | 19 | 0.54 |  | X | X |
| Alpha 3 type VI collagen | COL6A3 | 3 | 0.02 | Cancer | X | X |
| Alpha-2-glycoprotein 1, zinc | AZGP1 | 15 | 0.61 | Cancer |  |  |
| Alpha-2-HS-glycoprotein | AHSG | 11 | 1.71 | IR^d^, Cancer | X |  |
| Alpha-actinin-1 | ACTN1 | 21 | 1.70 |  |  | X |
| AMBP protein | AMBP | 6 | 0.45 | IR, Cancer | X |  |
| Apolipoprotein | LPA | 11 | 0.70 |  |  |  |
| Apolipoprotein C-I | APOC1 | 6 | 5.04 | Cancer | X | X |
| Apolipoprotein C-II | APOC2 | 9 | 1.97 |  |  |  |
| Apolipoprotein E | APOE | 25 | 2.07 | IR, Cancer | X |  |
| apolipoprotein F | APOF | 2 | 3.85 | Cancer | X |  |
| Apolipoprotein M | APOM | 3 | 1.50 |  | X |  |
| Argininosuccinate lyase | ASL | 2 | 0.62 |  | X |  |
| Beta-2-microglobulin | B2M | 5 | 0.61 | IR, Cancer | X | X |
| C4B1 | C4B | 128 | 1.50 | IR, Cancer | X |  |
| Cat eye syndrome critical region protein 1 | CECR1 | 2 | 8.40 |  |  |  |
| Cell surface glycoprotein MUC18 | MCAM | 2 | 1.79 | IR, Cancer |  |  |
| Cholinesterase | BCHE | 13 | 1.58 |  |  |  |
| Coagulation factor X | F10 | 9 | 0.69 |  | X |  |
| Complement component 4A | C4A | 126 | 1.29 | IR, Cancer | X |  |
| Complement component C8 alpha chain | C8A | 18 | 0.59 | IR | X |  |
| Complement factor D preproprotein | CFD | 4 | 0.20 | IR, Cancer | X |  |
| Contactin-1 | CNTN1 | 2 | 2.36 |  |  |  |
| C-reactive protein | CRP | 9 | 4.71 | IR, Cancer | X |  |
| Cystatin-C | CST3 | 3 | 1.57 | Cancer |  | X |
| EGF-containing fibulin-like extracellular matrix protein 1 | EFEMP1 | 4 | 0.53 | Cancer | X |  |
| Gelsolin | GSN | 44 | 0.58 | IR, Cancer |  |  |
| Insulin-like growth factor-binding protein 2 | IGFBP2 | 2 | 1.68 | Cancer | X | X |
| Inter-alpha-trypsin inhibitor heavy chain H1 | ITIH1 | 38 | 0.70 |  | X |  |
| Inter-alpha-trypsin inhibitor heavy chain H2 | ITIH2 | 62 | 0.70 |  | X |  |
| Phosphatidylinositol-glycan-specific phospholipase D | GPLD1 | 11 | 5.77 |  | X |  |
| Sulfhydryl oxidase 1 | QSOX1 | 2 | 3.13 |  |  | X |
| Kallistatin | SERPINA4 | 19 | 0.70 |  |  |  |
| Keratin, type I cytoskeletal 10 | KRT10 | 9 | 0.64 |  | X | X |
| Keratin, type II cytoskeletal 2 epidermal | KRT2 | 4 | 2.74 |  |  |  |
| L-lactate dehydrogenase B chain | LDHB | 9 | 2.08 |  |  | X |
| Lumican | LUM | 11 | 0.41 | IR |  | X |
| Mannan-binding lectin serine protease 1 | MASP1 | 5 | 0.57 | IR, Cancer | X |  |
| Mannose-binding protein C | MBL2 | 5 | 2.97 | IR |  |  |
| Metalloproteinase inhibitor 1 | TIMP1 | 2 | 0.21 | IR, Cancer | X | X |
| Moesin | MSN | 4 | 3.07 | IR |  | X |
| Monocyte differentiation antigen CD14 | CD14 | 7 | 1.5 | IR, Cancer |  | X |
| Peptidase inhibitor 16 | PI16 | 3 | 1.50 |  |  | X |
| Pigment epithelium-derived factor | SERPINF1 | 27 | 0.70 |  |  | X |
| Plasma protease C1 inhibitor | SERPING1 | 42 | 0.70 |  | X | X |
| Platelet basic protein | PPBP | 5 | 1.63 | IR |  | X |
| Poliovirus receptor | PVR | 2 | 0.56 | IR |  | X |
| Pregnancy zone protein | PZP | 12 | 2.15 |  |  |  |
| Prostaglandin-H2 D-isomerase | PTGDS | 3 | 0.06 | IR, Cancer | X |  |
| Protein disulfide-isomerase A3 | PDIA3 | 4 | 0.68 |  | X |  |
| Purine nucleoside phosphorylase | PNP | 2 | 0.53 | IR, Cancer |  | X |
| Rho GDP-dissociation inhibitor 2 | ARHGDIB | 4 | 0.13 | IR, Cancer | X | X |
| Selenium-binding protein 1 | SELENBP1 | 2 | 0.58 | Cancer |  |  |
| Selenoprotein P | SEPP1 | 3 | 0.70 |  | X | X |
| Semaphorin-7A /CD108 | SEMA7A | 2 | 1.76 | IR |  | X |
| Serum amyloid A | SAA2 | 8 | 1.75 |  | X | X |
| Serum amyloid A2 | SAA1 | 7 | 2.86 | IR, Cancer | X | X |
| Talin-1 | TLN1 | 40 | 2.15 |  |  | X |
| Transthyretin | TTR | 19 | 0.70 |  | X |  |

^a^ Ratio of the protein concentrations from HIV-infected patients with HL versus HIV-infected patients without HL.

^b^An “X” indicates that IPA lists these tissues or cell types as a known source of these proteins. There are commonly other tissues or cellular sources for these proteins that are not included in this table.

^c^WBC, white blood cells.

^d^IR, inflammatory response.
